# Prevalence and associated risk factors for depression symptoms in adolescent girls with polycystic ovary syndrome: a hospital-based cross-sectional study

**DOI:** 10.3389/fpubh.2024.1454415

**Published:** 2024-08-21

**Authors:** Lianhong Wang, Sihui Su, Tingting Xiong, Meili Wang, Rui Ding, Huiwen Tan, Minglan Zhu

**Affiliations:** ^1^Department of Nursing, Affiliated Hospital of Zunyi Medical University, Zunyi, Guizhou, China; ^2^Nursing School of Zunyi Medical University, Zunyi, Guizhou, China; ^3^Department of Obstetrics, Tongzi People's Hospital, Zunyi, Guizhou, China; ^4^Department of Reproductive Medicine, Affiliated Hospital of Zunyi Medical University, Zunyi, Guizhou, China; ^5^Department of Geriatric Medicine, The Third Affiliated Hospital of Zunyi Medical University, Zunyi, Guizhou, China

**Keywords:** depression symptoms, risk factors, polycystic ovary syndrome, adolescent girls, prevalence

## Abstract

**Background:**

Depression symptoms are a growing concern for adolescent girls with PCOS around the world. However, relatively small samples have given varying reports of its prevalence and risk factors in previous studies. Therefore, there is an urgent need for further research on the prevalence and associated factors of depression among adolescent girls with PCOS.

**Methods:**

A cross-sectional study was performed from October 2021 to May 2022 using a questionnaire and examination of the medical records of a convenience sample of 335 adolescent girls with PCOS. The Chinese version of the Children’s Depression Scale (CDI) was used to investigate depression symptoms. A multivariate logistic regression model was used to determine factors that were significantly associated with depression symptoms.

**Results:**

The prevalence of depression symptoms was 36.12% among adolescent girls with PCOS. A multivariate logistic regression model identified significant factors as perceived social support (95% CI: 0.921 ~ 0.965%, *p* = 0.000), sleep quality (95% CI: 1.134 ~ 1.324%, *p* = 0.000), belief illness (95% CI, 1.040 ~ 1.102%, *p* = 0.000), hirsutism (95% CI, 1.292 ~ 4.392%, *p* = 0.005), and LH/FSH ≥ 2 (95% CI, 1.939 ~ 6.369%, *p* = 0.000).

**Conclusion:**

Depression symptoms are an important problem among adolescent girls with PCOS in China. A comprehensive approach that encompasses social support, structured health education for the disease, and evaluation of the psychological status of PCOS girls with hirsutism (and) or LH/FSH ≥ 2 in time is important to minimize depression symptoms and improve psychological health among adolescent girls with PCOS.

## 1 Introduction

Polycystic ovary syndrome (PCOS) is a common endocrine metabolic disorder among women of reproductive age; the prevalence in adolescent girls is from 5.6 to 11.04% worldwide (1), and 10.26% of Chinese adolescent girls (2). Symptoms of PCOS include irregular menstruation, infertility, obesity, acne, hyperandrogenemia, and ovarian polycystic degeneration (3). PCOS also increases an individual’s risk for cardiovascular disease, type 2 diabetes, and endometrial cancer (4).

Simultaneously, PCOS is significantly related to mental illness. A previous study reported that adolescent girls with PCOS are 2.4 times more likely to suffer from depression than normal girls of the same age (5, 6). As a serious mental illness, depression presents with a depressed mood, decreased energy, loss of interest, feelings of guilt or low self-worth, disturbed sleep or appetite, and poor concentration (7). Depression has negative physical, psychological, social, and behavioral effects. In the adolescent population, more than 50% of suicides are related to depression (8, 9), and it also interferes with the successful transition from adolescence to adulthood: approximately 25% of adults suffer from depression during adolescence (10). As for PCOS, life management is recommended by international evidence-based guidelines as the ideal treatment. Patients with PCOS can participate in better life management by improving their psychological status. Therefore, depression is a significant health issue that must be addressed in the management of adolescents with PCOS (11).

As early as 2013, the PCOS Endocrinology Society’s clinical practice guidelines recommended identifying depression in adolescents with PCOS (5), and 2020 international evidence-based guidelines for adolescents with PCOS emphasize the need to manage depression (6). A number of previous studies showed the prevalence of depression symptoms in adolescent girls with PCOS.

However, in those studies, not only did the prevalence rate vary greatly, ranging from 12 to 60%, but the sample size was very limited; the largest number was 153 (12–15). As for the risk factors for depression among adolescents with PCOS, there were also very limited and inconsistent studies. According to Elsenbruch et al., (16) independent predictors of depression among adolescent girls were irregular or absent menstrual periods, infertility, the associated changes in appearance, and possible disturbances in sexual attitudes. Hopkins found that lower control was a predictor of greater depression among adolescent girls with PCOS (12). In the other two studies, there was no significant relationship between obesity, body mass index, acne, hirsutism, and insulin (INS) resistance and depression disorders in the PCOS group (17, 18).

Assessing the prevalence and associated factors of depression among adolescent girls with PCOS is important for early identification and intervention to reduce the effect of depression on adolescent girls with PCOS. Therefore, there is an urgent need for further research on the prevalence and associated factors of depression among adolescent girls with PCOS based on a large sample. Here, we enrolled 335 adolescent girls with PCOS and conducted an observational study to evaluate the prevalence of depression and comprehensively analyze the risk factors.

## 2 Methods

### 2.1 Design, setting, and participants

A hospital-based cross-sectional study design was used. Adolescent girls with PCOS were recruited from the gynecology clinic department of the affiliated hospital of Zunyi Medical University. This study was carried out from October 2021 to May 2022. The inclusion criteria include: (I) aged 10–19 years old (19); (II) Rotterdam diagnostic criteria recommended by the PCOS China Diagnosis and Treatment Guide in 2018 (20); (III) able to self-report (verbally understandable and articulate); and (IV) participants and their families volunteer to participate in the study. The exclusion criteria are as follows: (I) have other mental disorders and cognitive impairment; (II) have other serious diseases of important organs, and (III) have other diseases that lead to elevated androgen levels and ovulation disorders.

### 2.2 Sample size

A single population proportion formula $n={\left(Z\;\alpha /2\right)}^{2}p\left(1-p\right)/d^{2}$ was used to determine the sample size by taking the prevalence of depression among adolescent girls with PCOS as 39.2%, as assessed by the CDI (14), with a 5% margin of error, 95% confidence, and a 10% non-response rate. The final sample size was 312.

### 2.3 Data collection procedures

The researchers identified potentially eligible adolescent girls with PCOS who visited the gynecology clinic. If the adolescent girls with PCOS fulfilled the inclusion criteria, they and their parent(s) were briefed on the nature of the study and invited to participate after obtaining verbal consent from both the adolescent girls with PCOS and their parent(s).

Then two researchers begin to collect data on adolescent girls with PCOS according to the following three steps: the first step is a validated online self-administered questionnaire consisting of socio-demographic information, duration of PCOS, exercise habits, perceived social support, sleep quality, and belief illness was provided to adolescent girls with PCOS, and who needed to fill out the questionnaire independently. In the second step, researchers collected the height, weight, hirsutism, and acne of adolescent girls with PCOS in the gynecological clinic. In the third step, researchers collected laboratory indicators such as thyroid-stimulating hormone (TSH), luteinizing hormone (LH), follicle-stimulating hormone (FSH), total testosterone (TES), and INS from their medical records.

### 2.4 Instruments

#### 2.4.1 Depression symptoms

Our main outcome of interest was the provisional diagnosis of depression symptoms, which was assessed using the Chinese version of the CDI (21). The Chinese version of the CDI questionnaire has 27 items and 5 subscales of low self-esteem, negative affect, lack of pleasure, poor performance, and interpersonal problems. For each item, the response options range from 0 to 3, with 0 indicating the absence of symptoms, 1 indicating mild symptoms, and 2 indicating definite symptoms. The total scale score ranges from 0 to 54, with higher scores reflective of higher levels of depressive symptomatology, and a score of 19 indicated the presence of depressive symptoms. The total Cronbach’s alpha value was 0.85 in the reported study, and the test–retest reliability was 0.75.

#### 2.4.2 Perceived social support

Perceived social support was measured with the 12-item Perceived Social Support Scale (PSSS) (22). The scale yields three subscale scores for family, friends, and significant others. Respondents answered each question using a 7-point Likert scale ranging from 1 = “very strongly disagree” to 7 = “very strongly agree.” Higher scores indicate that subjects perceive more social support and are categorized as low (12–36), moderate (37–60), or high (61–84). The total Cronbach’s alpha value was 0.927 in the reported study.

#### 2.4.3 Sleep quality

Sleep quality was measured using an 8-item of Athens Insomnia Scale (AIS) (23). For each item, the response options range from 0 to 3, with 0 indicating no problem at all and 3 indicating serious problems. The total scale score ranges from 0 to 24, with higher scores reflecting lower levels of sleep quality; a score of 6 indicates poor sleep quality. For internal consistency, Cronbach’s alpha was approximately 0.90, and the mean item-total correlation coefficient was approximately 0.70.

#### 2.4.4 Belief illness

Belief illness was measured using a 9-item Brief Illness Perception Questionnaire (BIPQ) (24).For each item, the response options range from 0 to 10, and the total scale score ranges from 0 to 80, with higher scores reflective of higher levels of negative perception of disease. The questionnaire had an internal consistency coefficient of 0.831, and the test–retest reliability was 0.931 in the reported study.

#### 2.4.5 Hirsutism

The modified Ferriman–Gallwey scoring system was used for the diagnosis of hirsutism (25). These areas included the upper lip, lower back, lower abdomen, and thigh. Values between 0 (no excess hair growth) and 4 (extremely disturbing hair growth) in patients may be evaluated. The total scale score ranges from 0 to 16, and patients with FG scores of 3 or higher are regarded as hirsute.

#### 2.4.6 Acne

Acne was graded using the Rosenfield scoring system (26), which may be classified as mild, moderate, or severe based on the number, extent, and nature of the skin lesions and consists of six items with values between 0 (= no acne) and 5 (= cystic acne). The total scale score ranges from 0 to 5, with higher scores indicating more severe disease.

### 2.5 Statistical analysis

Data entry and analysis were performed using EpiData 3.1 and SPSS - version 21. Descriptive statistics were presented as percentages (*n*, %) and mean ± SD for continuous variables. We analyzed the differences in characteristics of PCOS girls with or without depression symptoms using bivariate logistic analysis, in which independent t-tests or chi-square tests were conducted. All independent variables with a *p*-value of <0.05 in the bivariate logistic analysis were candidates to enter the multivariable logistic regression model to control the confounding effect. A multivariable logistic regression model was used to identify factors associated with depression symptoms among PCOS girls, with a *p*-value of <0.05 with 95% CI was considered statistically significant.

## 3 Results

A total of 335 adolescent girls with PCOS were recruited for this study through convenience sampling, and all of the data were collected. This left an effective response rate of 100%.

### 3.1 Participant characteristics

Of the 335 adolescent girls with PCOS who were enrolled in the assessment, we have reported that 121 adolescent girls with PCOS were screened positive for depression symptoms with CDI at a cutoff point of 19, thus resulting in a prevalence of 36.12%.

Of the 335 adolescent girls with PCOS, the mean age was 17.66 ± 1.657 years old (ranging from 12 to 19 years old). The majority (60.60%) of the adolescent girls with PCOS included in this study were from rural, 179 (53.43%) had an education level of senior high school, the mean duration of the disease diagnosis was 6.2 ± 5.7 years, 232 of the adolescent girls with PCOS (69.25%) had no regular exercise, the mean BMI was 23.257 ± 4.426, 109 (31.0%) and 80 (23.89%) had hirsutism and acne, respectively. Of all adolescent girls with PCOS, 51.94% had the LH/FSH ≥ 2. Analysis of participant characteristics is shown in Table 1.

**Table 1:** Characteristics of PCOS girls with and without depression symptoms (*n* = 335).

Factors	PCOS girls with depression symptoms (*n* = 121)	PCOS girls without depression symptoms (*n* = 214)	*x^2^/t*	*p*
	*n* (%) M ± SD	*n* (%) M ± SD		
Age	17.77 ± 1.559	17.60 ± 1.711	0.879^■^	0.380
Residence *n* (%)			0.025^▲^	0.875
Rural	74 (61.16%)	129 (60.28%)		
Urban	47 (38.84%)	85 (39.72%)		
Educational level *n* (%)			0.721^▲^	0.890
Junior high school or less	10 (8.27%)	24 (11.21%)		
Senior high school	66 (54.54%)	113 (52.81%)		
College or more	45 (37.19%)	77 (35.98%)		
The only child *n* (%)			0.583^▲^	0.445
Yes	25 (20.66%)	37 (17.29%)		
No	96 (80.17%)	177 (82.71%)		
Single parent family *n* (%)			0.645^▲^	0.422
Yes	24 (19.83%)	35 (16.36%)		
No	97 (80.17%)	179 (83.64%)		
Family history of mental illness *n* (%)			0.234^▲^	0.628
Yes	9 (7.44%)	13 (6.07%)		
No	112 (92.56%)	201 (93.93%)		
Smoking status *n* (%)			1.403^▲^	0.528
Never smoked	110 (90.91%)	201 (93.93%)		
Ex-smoker	6 (4.96%)	6 (2.80%)		
Current smoker	5 (4.13%)	7 (3.27%)		
Drinking status *n* (%)			0.230^▲^	0.936
Never drink	114 (94.21%)	200 (93.46%)		
Ex-drinker	3 (2.48%)	5 (2.34%)		
Current drinker	4 (3.31%)	9 (4.20%)		
Regular exercise (≥3 times/week, ≥30 min per session) *n* (%)			0.876^▲^	0.349
Yes	41 (33.88%)	62 (28.97%)		
No	80 (66.12%)	152 (71.03%)		
Duration of PCOS	3.02 ± 1.446	2.82 ± 1.516	1.193^■^	0.234
Take hormonal contraception medicines *n* (%)			10.158^▲^	0.001
Yes	104 (85.95%)	140 (65.42%)		
No	17 (14.05%)	74 (34.58%)		
Perceived social support	52.65 ± 10.96	65.76 ± 15.83	−7.046^■^	<0.001
Sleep quality	9.71 ± 9.71	6.10 ± 3.71	8.538^■^	<0.001
Brief illness	50.09 ± 9.18	41.42 ± 11.14	7.679^■^	<0.001
BMI	23.33 ± 4.54	23.13 ± 4.30	0.402^■^	0.688
Hirsutism			9.401^▲^	0.002
Yes	52 (43.0%)	57 (26.6%)		
No	69 (57.0%)	162 (73.4%)		
Acne			0.315^▲^	0.575
Yes	31 (25.6%)	49 (22.9%)		
No	90 (74.4%)	165 (77.1%)		
TES	1.51 ± 0.77	1.4 ± 0.9	0.659^■^	0.511
INS	11.07 ± 7.02	10.50 ± 8.01	0.663^■^	0.508
PRL	357.08 ± 166.49	356.87 ± 191.87	0.010^■^	0.992
LH/FSH ≥ 2			23.189^▲^	<0.001
Yes	84 (69.4%)	90 (42.1%)		
No	37 (30.6%)	124 (57.9%)		

PCOS, Polycystic ovary syndrome; M ± SD, mean ± standard deviation; *x^2^*, chi-square tests; *t*, independent t-tests; BMI, body mass index; TES, total testosterone; PRL, prolactin; INS, insulin; LH, luteinizing hormone; FSH, follicle-stimulating hormone; ^■^, t-test; ^▲^, *x*^2^.

### 3.2 Factors associated with depression symptoms in adolescent girls with PCOS

In the univariate analysis, six factors were found to be significantly associated with depression symptoms in adolescent girls with PCOS, including having hormonal contraception medicines (*p* = 0.001), perceived social support (*p*<0.001), belief illness (*p* = 0.001), sleep quality (*p* = 0.001), hirsutism (*p* = 0.002), and LH/FSH ≥ 2 (*p*<0.001) (Table 1).

The multivariable logistic regression model analyses identified five statistically significant factors associated with being more likely to develop depression symptoms in adolescent girls with PCOS: perceived social support (OR = 0.944, 95% CI =0.917 ~ 0.971), sleep quality (OR = 1.246, 95% CI =1.131 ~ 1.374), belief illness (OR = 1.082, 95% CI =1.042 ~ 1.123), Hirsutism (OR = 2.530, 95% CI =1.190 ~ 5.377), and LH/FSH ≥ 2 (OR = 2.858, 95% CI =1.400 ~ 5.836) (Table 2).

**Table 2:** Factors associated with depression symptoms among adolescent girls with PCOS.

Covariates	*B*	S.E	Wald	*p*	OR	95%CI
Take hormonal contraception medicines *n* (%)	0.726	0.426	2.896	0.089	2.066	0.896 ~ 4.766
Perceived social support	−0.059	0.012	25.026	0.000	0.943	0.921 ~ 0.965
Sleep quality	0.203	0.039	26.583	0.000	1.225	1.134 ~ 1.324
Belief illness	0.068	0.015	21.002	0.000	1.070	1.040 ~ 1.102
Hirsutism	0.868	0.312	7.739	0.005	2.382	1.292 ~ 4.392
LH/FSH ≥ 2	1.257	0.303	17.167	0.000	3.515	1.939 ~ 6.369

LH, Luteinizing hormone; FSH, follicle-stimulating hormone.

S.E, Standard error; OR, odds ratio; CI, confidence interval.

Our study used the Box–Tidwell method to verify whether the continuous independent variable and the dependent variable logit conversion values were linear. The linear test model included six variables: hormonal contraception medicines, sleep quality, perceived social support, hirsutism, belief illness, and LH/FSH ≥ 2). After the Bonferroni correction, the significance level was 0.005. The line test results showed a linear relationship between all continuous independent variables and the dependent variable logit conversion values. Finally, the resulting logistic model was statistically significant (*x*2 = 114.098, *p* < 0.001). A total of five variables associated with depressive symptoms in PCOS patients were statistically significant in the model, including hirsutism (OR = 2.530, 95%CI: 1.190–5.377, *p* = 0.016), LH/FSH ≥ 2 (OR = 2.858, 95%CI: 1.400–5.836, *p* = 0.004), perceived social support (OR = 0.944, 95%CI: 0.917–0.971, *p*<0.001), sleep quality (OR = 1.246, 95%CI: 1.131–1.374, *p*<0.001), and belief illness (OR = 1.082, 95%CI: 1.042–1.123, *p*<0.001).

## 4 Discussion

The prevalence of symptoms of depression was 36.12% among adolescent girls with PCOS in China. Our research finds that six factors (such as social support, sleep quality, belief illness, hirsutism, and LH/FSH ≥ 2) play a critical role in depression symptoms among adolescent girls with PCOS.

To our knowledge, this was the first study with respect to the depression symptoms of adolescent girls with PCOS in China. In the present study, the 36.12% prevalence rate of depression symptoms is in line with a study conducted in Turkey at 39.2% (14), and the reasons for the phenomenon may be related to using the same CDI and the participants were all from the gynecology clinic department of a general hospital. However, the current finding is lower than two small sample studies conducted in America (*n* = 47 and *n* = 23), which found the prevalence of depression among adolescent girls with PCOS to be 60 and 56.5%, respectively (12, 13). On the contrary, the present study’s finding is higher than a study conducted in New Zealand, which found the prevalence of depression symptoms among adolescent girls with PCOS to be only 12% (15). The variations might be due to differences in environmental factors, sample size, a tool for assessing depression, and multiple sources of participants.

According to this study, adolescent girls with PCOS who have lower levels of social support have a higher risk of developing depression. The finding corroborates the result of a previous study which was conducted in America (12). This may be explained as follows: PCOS is a common chronic disease, but public awareness and attention to this disease are insufficient. As a result, adolescent girls with PCOS are more likely to have a sense of isolation, increasing the risk of depression.

In the present study, we found that sleep quality is associated with depression symptoms among adolescent girls with PCOS. A systematic review and meta-analysis of cohort studies reported that there is an association between sleep disruption and depressive symptoms in children and youths (27). Another systematic review of the literature regarding sleep duration and mood in adolescents indicated that less sleep was associated with a 55% increase in the likelihood of mood deficits (28).

Different mechanisms have been proposed to explain the association between sleep problems and depression, such as a disruption of circadian rhythm, activation of inflammatory pathways, and altered neuroplasticity and learning (29), a reduction in prefrontal activity, and a decrease in functional connectivity between the prefrontal cortex and limbic regions (30), and the loss of a person’s ability to shift attention away from repeated negative thoughts (31).

Our study demonstrated that belief illness was associated with depression symptoms among adolescent girls with PCOS. Similar to our findings, perceived stress of the disease was an independent risk factor for depression in women with PCOS (32). Many adolescent girls with PCOS reported being uncertain about many aspects of PCOS, and a study indicated that PCOS patients with higher negative perceptions of the disease and its clinical manifestations were more pronounced (33).

Body image is a multidimensional matter that includes the perception of people regarding self-appearance and related thoughts and feelings about it. Hirsutism, acne, and obesity as important influence factors of body image are common symptoms among women with PCOS. Our study found there was a positive association between hirsutism and depression symptoms in adolescent girls with PCOS. Many studies also found that hirsutism was an independent risk factor for depression symptoms in women with PCOS (33–37). The possible reason could be, first hirsutism is characterized by excessive terminal hair growth in a male—pattern distribution in female patients, including the face, chest, back, upper arms, thighs, and abdomen. The unwanted facial and bodily hair often led to the feeling of “not being a female,” which may be theorized to contribute to the psychological burden in this population of relatively young women. Second hirsutism is one of the most important manifestations of high androgen levels, which has a close relationship with depression (38, 39). Contrary to previous findings, which showed that there was a clear association between higher depression scores and elevated BMI among adolescent girls with PCOS (15). In the present study, we found no significant relationship between BMI and depression. This might be a result of the different sample sizes; in the present study, the sample size is 335, which is three times more than in the previous study.

In the present study, we found that LH/FSH ≥ 2 is significantly correlated with depression symptoms in adolescent girls with PCOS. This is consistent with the study by Feng et al., who reported that LH/FSH ≥ 2 is an independent risk factor for depression in patients with PCOS. The mechanism for this phenomenon is with the increase of LH/FSH ratio the incidence of menstrual disorders increased (40). Studies reported that there was a positive association between menstrual disorders and depression symptoms (41, 42).

## 5 Limitations

Some limitations must be considered in the interpretation of these findings. First, although in our study we enrolled 335 adolescent girls with PCOS, which was relatively a larger sample size compared to the previous studies, it is not possible to generalize the results of the study considering that the sample was recruited in a single hospital. Second, it was not randomized but a convenience sample. The other limitations might be that the design of the study was cross-sectional, and temporality and causality between factors could not be assured.

## 6 Conclusion

In summary, approximately 36.12% prevalence of depression symptoms was observed among adolescent girls with PCOS in China. Moreover, our research finds that many factors (such as social support, sleep quality, belief illness, hirsutism, and LH/FSH ≥ 2) play a critical role in depression symptoms among adolescent girls with PCOS. Our results suggest the importance of a comprehensive approach at both individual and institutional levels to reduce depression symptoms among adolescent girls with PCOS. More specifically, a holistic strategy that encompasses social support (such as emotional and informational support from parents, peers, and medical staff), structure health education for the disease, and evaluation of the psychological status of PCOS girls with hirsutism (and) or LH/FSH ≥ 2 in time.

## Data availability statement

The raw data supporting the conclusions of this article will be made available by the authors, without undue reservation.

## Ethics statement

The studies involving humans were approved by the Ethics Committee of Zunyi Medical University: Zunhe Lun Review [2021] 1–093. The studies were conducted in accordance with the local legislation and institutional requirements. Written informed consent for participation in this study was provided by the participants’ legal guardians/next of kin.

## Author contributions

LW: Conceptualization, Formal analysis, Supervision, Validation, Writing – original draft, Writing – review & editing. SS: Data curation, Investigation, Writing – review & editing. TX: Data curation, Investigation, Writing – review & editing. MW: Conceptualization, Formal analysis, Visualization, Writing – review & editing. RD: Data curation, Investigation, Writing – review & editing. HT: Data curation, Investigation, Writing – review & editing. MZ: Conceptualization, Formal analysis, Project administration, Supervision, Writing – review & editing.
